# The power comparison of the haplotype-based collapsing tests and the variant-based collapsing tests for detecting rare variants in pedigrees

**DOI:** 10.1186/1471-2164-15-632

**Published:** 2014-07-28

**Authors:** Wei Guo, Yin Yao Shugart

**Affiliations:** Division of Intramural Division Program, National Institute of Mental Health, National Institute of Health, 35 Convent Drive, Bethesda, MD 20892 USA

**Keywords:** Haplotype, Pedigree disequilibrium test, Rare variants, Weights, Association

## Abstract

**Background:**

Both common and rare genetic variants have been shown to contribute to the etiology of complex diseases. Recent genome-wide association studies (GWAS) have successfully investigated how common variants contribute to the genetic factors associated with common human diseases. However, understanding the impact of rare variants, which are abundant in the human population (one in every 17 bases), remains challenging. A number of statistical tests have been developed to analyze collapsed rare variants identified by association tests. Here, we propose a haplotype-based approach. This work inspired by an existing statistical framework of the pedigree disequilibrium test (PDT), which uses genetic data to assess the effects of variants in general pedigrees. We aim to compare the performance between the haplotype-based approach and the rare variant-based approach for detecting rare causal variants in pedigrees.

**Results:**

Extensive simulations in the sequencing setting were carried out to evaluate and compare the haplotype-based approach with the rare variant methods that drew on a more conventional collapsing strategy. As assessed through a variety of scenarios, the haplotype-based pedigree tests had enhanced statistical power compared with the rare variants based pedigree tests when the disease of interest was mainly caused by rare haplotypes (with multiple rare alleles), and vice versa when disease was caused by rare variants acting independently. For most of other situations when disease was caused both by haplotypes with multiple rare alleles and by rare variants with similar effects, these two approaches provided similar power in testing for association.

**Conclusions:**

The haplotype-based approach was designed to assess the role of rare and potentially causal haplotypes. The proposed rare variants-based pedigree tests were designed to assess the role of rare and potentially causal variants. This study clearly documented the situations under which either method performs better than the other. All tests have been implemented in a software, which was submitted to the Comprehensive R Archive Network (CRAN) for general use as a computer program named rvHPDT.

## Background

Genetic studies such as genome-wide association studies (GWAS) have typically relied on the common disease/common variant (CDCV) paradigm, and the GWAS approach has had its share of success with some genetic disorders. Although earlier analyses of GWAS data revealed that this approach can detect common variants with modest effects, however, only a small portion of these significant associations with common variants were subsequently found to be functional and these associations account for a small portion of the total heritability of genetic variations. On the other hand, in recent years, the role of rare genetic variants has received more attention due to the advent of next generation sequencing (NGS) technology. Indeed, some rare variants were found to play a causal role in human diseases, including psychiatric disorders. This led to the shift from the CDCV approach to a common disease-rare variant (CDRV) approach.

Given this increased recognition that common disorders may in fact reflect the aggregated effects of many rare variants, genetic analysts investigating this new hypothesis have had to deal with a host of new challenges. The more affordable whole genome sequencing (WGS) technology demands new analytical tools to analyze these data.

Many methods have been developed to analyze data from case–control studies. These include: 1) collapsing methods [1]; 2) the weighted-sum association method [2, 3]; 3) pooled association tests for rare variants [3]; 4) the aSum test [4, 5]; 5) the alpha test [6]; and 6) the sequencing kernel association test (SKAT) [7]. The interested reader is referred to three thoughtful papers that review current methods for collapsing and pooling data [8, 9]. Family based studies can be used to address the issue of rarity via the enrichment of rare variants within pedigrees. One impediment to family-based sequencing, however, is the fact that, with few exceptions, the statistics used to study rare variants were traditionally designed for use with population-based data. Family-based approaches include *FBAT* software (v2.0.4), which provides both un-weighted (*FBAT-v0*) and weighted sum tests (*FBAT-v1*) to analyze rare variants [10]. The *FBAT-v1* test is weighted based on allele frequency, similar to the weighted sum statistic (WSS) [2]. Shugart and colleagues [11] proposed weighted pedigree-based statistics using the function of kinship matrix as a correction factor to adjust family correlations. And Zhu and Xiong [12] proposed a sophisticated family-based functional principal-component analysis (FPCA) with or without smoothing. On the quantitative traits side, Guo and Shugart [13] used beta-determined weight testing to analyze nuclear families, and collapsed rare variants using regression coefficients. More recently, Chen and colleagues [14] proposed using SKAT to analyze rare variants in family samples.

It is worth noting that all the family-based association tests listed above are variant-based approaches. Nevertheless, haplotypes—which are combinations of alleles (namely, the DNA sequences) at adjacent locations on a chromosome—has the potential to provide further insight into the structure of underlying linkage disequilibrium (LD), thus potentially yielding higher power in association studies investigating common and rare variants [15, 16]. For certain complex diseases such as hypertension, rare haplotypes have been shown to influence disease susceptibility [17–19]. Here, to highlight the importance of haplotype-based approaches, we provide a brief review on the studies detected haplotypes composed of multiple rare variants contributing to disease or traits related to lipid metabolism. In 2002, Knoblauch and colleagues [20] reported a robust haplotype effect in six genes including the cholesterol ester transfer protein (CETP), Lipoprotein (LPL), hepatic triglyceride (HL), low-density lipoprotein cholesterol receptor (LDLR), Apolipoprotien E (ApoE) and lecithin-cholesterol acyltransferase (LCAT) on lipid concentrations. More specifically, the authors recruited 732 individuals from 184 German families and identified 26 genetic variants (a combination of rare and common variants) in CETP, LCAT, HL, LPL and LDLR. They utilized the family structure to help establish the haplotypes and observed a haplotype effect on the genetic variance of the LDL/HDL ratio [20]. Later, Cohen et al. [21] documented the contribution by multiple rare variants to low plasma levels of high density lipoprotein cholesterol (HDL-C) after sequencing the coding regions and consensus splice sites of genes ATP-binding cassette transporter (ABCA1), Apolipoprotein (APOA1) and Lecithin (LCAT) in 256 individuals of the Dallas Heart Study (DHS). Here we highlight the findings in the ABCA1 gene as a successful example. Cohen et al. examined the sequence variants which are unique to the low HDL-C group and the high HDL-C group and identified 14 non-synonymous mutations in the low HDL-C group, and 6 synonymous mutations in the high HDL-C group. Similar observations on causal rare variants were made in a Canadian sample (155 with low HDL-C and 108 with high HDL-C). For the non-synonymous genes, the authors reported the predicted effects for some of the amino acid changes. A majority of them were predicted to be “possibly damaging”. Although the authors did not use a statistical framework to examine this work, their results suggested an example for a gene-based haplotype with a number of rare causal and non-causal variants. In 2007, Saleheen et al. [22] reported a novel haplotype in ABCA1 gene associated with plasma HDL-C concentration. The authors selected five non-synonymous single nucleotide polymorphisms (SNPs) after sequencing ABCA1 in 200 unrelated Pakistanis individuals of who are free of ischemic heart diseases. Furthermore, they aimed at detecting causal mutation in individuals having arterial hypertension. In their study, R219K, V399A and V771M polymorphisms were not associated with either HDL-C or LDL-C. On the other hand, their haplotype analysis revealed a signal for an interaction between R219K and V825L polymorphisms. Further, the RL haplotype was found to be associated with the low levels of HDL-C [*P* = 0.001]. More recently, Slatter et al. [23] reported five rare mutations (in five of the low-HDL samples) and promoter haplotypes in ABCA1 in 154 low-HDL samples and 102 high-HDL samples. More specifically, their analysis of four SNPs in ABCA1’s promoter region identified the over-representation of the “C-14 T” SNP and the “TCCT” haplotype in the low-HDL individuals [23]. Together, all these studies indicated the clinical importance of haplotypes composed of multiple rare variants.

At the theoretical level, Lin and colleagues [8] pointed out that haplotypes may serve as the “superalleles” that combine the joint effects from uncommon causal variants not genotyped in GWAS. While the research community has accepted the concept of haplotype-based analysis, to our knowledge the use of haplotype-based tests as tools to detect rare variants in pedigree data remains unstudied. Here, we review several pedigree-based statistics specifically designed to test for rare variants in family-based data and then introduce our new methods—the haplotype-based pedigree tests—that uses the haplotype constructed by rare or uncommon variants. In general pedigrees, PDT [9], which breaks a pedigree into case-parent trios and discordant sib-pairs, and then sum up their contributions, has been often used in the field of genomic research. Using this approach, rare haplotypes are collapsed under the framework of the PDT in pedigree data.

Overall, we developed five tests termed *hPDT*, *maxH*, *maxV*, *hPDT-t* and *vPDT-t*. The detailed mathematical expressions will be provided under Methods. Our main goal is to investigate the performance of haplotype and variants based tests in general pedigrees, which will allow us to address the question of whether or not haplotype-based collapsing tests would outperform the variant-based collapsing tests for detecting rare variants in human pedigrees under difference cases of scenarios.

## Methods

### The hPDT and vPDT

Let *N* be the total number of pedigrees. Let *M* be the total number of variants in the gene of interest, and *H* haplotypes formed are denoted by *h*_1_, *h*_2_, …, *h*_*H*_. For the pedigree *i*, let *n*_*T*_ be the number of informative nuclear families and *n*_*S*_ be the number of informative discordant sibships. Similar to PDT, *hPDT* considers the difference in the number of transmitted and untransmitted haplotypes from parents to affected siblings and the difference in the number of haplotypes between affected and unaffected siblings. For *k-*th haplotype, the *hPDT* statistic for pedigree *i* is thus defined as:
1

Where  is the number of transmissions minus the number of non-transmissions on the *h* th haplotype in trio *j*, and  is the number of copies in the affected sib minus the number of copies in the unaffected sib in sib-pair *j*.

Without loss of generality, assume *h*_*H*_ is the most frequent haplotype, and for the rest of *H* − 1 haplotypes, the summary random variable for the pedigree *i* is defined as:
2

where *w*_*k*_ is the weight for the haplotype *k*. We define a similar weight function as in Madsen and colleagues [2]:
3

where *λ* is the number of individuals in *N* families, and *q*_*h*_ is the haplotype frequency of the *k*-th haplotype. Under the null hypothesis of no association,  for all trios and  for all discordant sib pairs and, consequently,  and *E*(*D*_*i*_) = 0 for any pedigree structure. If *N* families are unrelated, under the null hypothesis of no association
4

and
5

The above derivation is adopted from the material provided by the original authors of the PDT test [9].

For *N* unrelated families, the *hPDT* statistic is defined as:
6

which asymptotically follows a standard normal distribution under the null hypothesis of no association based (see Equation (5)).

In spirit, the *vPDT* test (Chung R, Tsai H, Eu B, Kao H, Chiu Y: Extension of the pedigree disequilibrium test for rare variants: an application to the genetic analysis workshop 18 dataset. Unpublished) is similarly defined as the *hPDT* test; however *vPDT* simply combines the *M* rare variants using allelic frequencies determined weights while the *hPDT* test use haplotype frequencies determined weights.

### The maxH and maxV

For *k*-th haplotype, the *PDT* statistic could be constructed as
7

and the *maxH* test is defined as the maximal value of *Z*^(*k*)^ for all haplotypes except for the most frequent one. Similarly, the maximal variants test, *maxV*, could be constructed by the maximal value of PDT tests based on single variant over all *M* rare variants. The p-values of *maxH* and *maxV* are obtained by a standard permutation procedure. For the *maxH* test, the transmitted and non-transmitted haplotypes of each offspring are randomly sampled from the parental haplotypes; for the *maxV* test, the transmitted and non-transmitted alleles of each child are randomly sampled from the parental alleles on each rare variant.

For a single analysis sample (i.e., for a real data analysis), we propose to adopt the standard permutation procedure for obtaining p-values of *maxH* and *maxV* tests. For simplicity, we assume *T* is the test statistic of interest, and *T* is either *maxH* or *maxV* here. Specifically, we permute the transmission status (transmitted and non-transmitted) *B* times to obtain *T*_*b*_, *b* = 1, …,*B*. These can be regarded as a sample of size *B* from the distribution of *T* (as defined in Equation (7)) under the null hypothesis. Then
8

provides a permutated p-value for the *maxH* and *maxV* tests. For a simulation study with *R* replicates, the above procedure will be time-consuming. Therefore, we propose the following steps to reduce the computational burden by pooling permutation samples from all replicates to form a joint sample from the null distribution. More specifically, for the *r*th replicate with observed test statistic *T*_*r*_, we permuted the labels *B* times as above to obtain *T*_*r,b*_, *b* = 1, …,*B*. Then the collection {*T*_*r,b*_; *r* = 1, …,*R*, *b* = 1, …,*B*} can be regarded as a random sample of size *B × R* from the common null distribution, i.e., the distribution under the null hypothesis *H*_0_. Consequently, for *r* = 1, …,*R*,
9

provides the permuted p-value for the *r*th replicate. Since the permutation samples are pooled across all replicates to form a sample from the null, *B* can be set to be much smaller than the situation when only one sample is analyzed. For example, if *R* = 1000 and the desired number of permutations for estimating the p-values is 10000, then we set *B* = 10 for each replicate. In other words, we do not need to perform 10000 permutations for each replicate to obtain the p-value. Instead, we only need to perform 10 permutations per replicate to achieve the same precision level. This pooling strategy is similar to what was proposed in Becker and Knapp [24].

### hPDT-t and vPDT-t

To adapt to the case of scenarios that both risk haplotypes (increasing risks) and protective haplotypes (decreasing risks) exist, one may benefit from estimating the direction of each haplotype before the weights are defined using Equation (3). To accomplish this task, the data set is randomly split into a testing set and a training set. In our analysis, 30% of data is selected as the training set and the other 70% of data is remained as the testing set. In the first step, the single-haplotype tests are carried on within the training set, and then new weights are defined as:
10

where *Z*^(*k*)^ is the *PDT* statistic for *k*-th haplotype in the training set defined as Equation (7) and *λ* and *q*_*k*_ are identically defined as in Equation (7) using all data. For example, *μ* = 1.04 (1.28 and 1.64) corresponds to a type I error of 0.3 (0.2 and 0.1, respectively). We set *μ* = 1.04 to preserve more nonzero weights/haplotypes in the next step tests. In the second step, the *hPDT-t* statistic is defined as Equation (6) within the test set.

The *vPDT-t* is similarly defined as the *hPDT-t* test by replacing haplotypes by variants.

### Infer haplotypes of pedigree members

For the unphased genotype data, haplotypes were inferred by MERLIN [25] software, which is commonly used to reconstruct putative haplotypes for families and individuals using likelihood-based methods.

For any ambiguous loci that cannot be phased using the available information, MERLIN software provides output for all possible patterns of gene flow. To resolve potential haplotyping ambiguity caused by incomplete phasing and missing variants, an EM algorithm is employed to estimate haplotype frequencies and family weights for each gene flow simultaneously, where the sum of family weights is one for a single pedigree. For *hPDT* and *hPDT-t*, the statistics in Equation (1) were modified as  where *Weight*^(*f*)^ is the weight for the *f* th gene flow while  is the normal *D*_*i*_ statistic defined by Equation (2).

## Results and discussion

### Simulation

For the purpose of simulation, the forward evolutionary simulation tool *ForSim* [26] was used to simulate genotypic data within pedigrees. *Forsim* can simulate pedigrees and take evolutionary processes—such as natural selection, mutation, and population demographics—into account. While running *Forsim*, the mutation rate is assumed to be 2.5 × 10^− 8^, the total number of generations is set at 500, the recombination rate is 1 cM per Mb, the growth rate is 0.525, the fertility Poisson mean is 8, the maximal number of children is 500, the initial size of the population is 1000, and the carrying capacity is 20,000. The gene length can also be fixed at 1 Mb and chromosome length at 5 Mb. Because we re-simulated the phenotype values, other parameters about the disease model did not matter in *Forsim* and had no influence in this second simulation. To evaluate the performance of the *hPDT*, we retained only the pedigree structure and genotype data simulated using *Forsim*, and re-simulated the affected status for each individual using newly assumed disease models (described in detail later). Two hundred nuclear families were randomly selected, and each family included two parents, two affected siblings, and one unaffected sibling. The total numbers of individuals were 1000.

To assign affected status we followed a disease model similar to that described by Li and colleagues [16]. Within the 1 Mb gene region generated in a 5 Mb genome by *Forsim*, we randomly picked 20 Kb regions as the causal region; only rare variants were to be tested in our simulation within this causal region. Here, rare variants were defined as “rare” when its MAF was between 0.001 and 0.05.

To investigate type I error rates, we set the penetrance at 30% in order to obtain a sufficient number of pedigrees with affected members. Disease status was assigned according to *P*(affected|{*h*_1_, *h*_2_}) = 30 % when simulating phenotype data under the null hypothesis.

To evaluate the power, we randomly selected *d%* (*d%*=50%, 40%, 30%, 20%, and 10%) of the rare variants in the region of interest to be causal. Then, among these rare variants, we further assumed that *r%* (*r%*=100%, 80%, 50%, 20%, and 5%) of them increased disease risk, whereas the remaining *(100 – r)*% decreased disease risk. Following methods outlined by Madsen and Browning [2], we assumed that the less frequent variant made a greater contribution to disease, and that the contribution of each causal variant *l* to the overall genotype relative risk (GRR) was defined as:
11

where *PAR* is the population attributable risk set as 0.006 [16]. For an individual with a pair of haplotypes {*h*_1_, *h*_2_}, the disease status was assigned according to:
12

in which *f*_0_ was the baseline penetrance and was fixed at 10% in our simulations, *M*_*c*_ was the number of causal SNPs among *M* SNPs in the region, *h*_*k*,*l*_ was the *l* th causal allele in haplotype *h*_*k*_, and *a*_*l*_ was the rare allele of SNP *l*.

### Special haplotype setting for investigating effects of LD

In keeping with the method of Li and Leal [1], we also investigated the effects of LD using simulation. The following steps were taken: 10 variants were considered. Fifteen haplotypes were constructed as follows: 1010000000, 0110000000, 1001000000, 0101000000, 1000000000, 0100000000, 0010000000, 0001000000, 0000100000, 0000010000, 0000001000, 0000000100, 0000000010, 0000000001, 0000000000, where 1 indicates the minor allele and 0 indicates the major allele. While the first fourteen rare haplotypes have equal frequencies of 0.01, the last common haplotype has frequency of 0.86. The MAFs of the first four variants are 0.03, and the MAFs of remain six variants are 0.01. Let  be the *R*^2^ values (*R*^2^ is a measurement of LD and defined as squared correlation coefficient between pairs of loci) between variant *i* and variant *j*, where *i*, *j* = 1, …, 10. There were four pair-wise LD  while other pair-wise LDs are close to zero (<0.0005). To generate the data, parental haplotypes were randomly assigned according to the assumed haplotype frequency. The parental haplotypes were then transmitted to offspring without recombination.

Finally, the affection status of an individual with *h*_*k*1_*h*_*k*2_ haplotype in the pedigree was assigned to be a random variable according to the following trait model, logit{*P*(affected|*h*_*k*1_*h*_*k*2_)} = *β*_*k*1_ + *β*_*k*2_, where the *logit* function is defined as , and *k*_1_ and *k*_2_ (*k*_1_, *k*_2_ = 1, …, 15) indicate 15 haplotypes defined as above. *β*_*k*_ (*k* = 1, …, 15) indicate the effect sizes in trait model, and *β* s were specified as (1) *β*_1_ = *β*_4_ = 3, *β*_5_ = *β*_6_ = *β*_7_ = *β*_8_ = − 3; (2) *β*_1_ = *β*_4_ = 3, *β*_5_ = *β*_6_ = 1, *β*_7_ = *β*_8_ = − 3; (3) *β*_1_ = *β*_2_ = *β*_3_ = *β*_4_ = 2, and other *β*_*s*_ were zero in each setting. Under this three β settings, the cumulative effect sizes (*β*_*s*_) on ten rare variants were (0,0,0,0,0,0,0,0,0,0), (4,3,0,0,0,0,0,0,0,0) and (4,4,4,4,0,0,0,0,0,0), respectively.

## Results

Type I error rates and analytical power associated with six tests were assessed using the nuclear families simulated both by *forsim* and by the direct simulation under the special haplotype setting. Six tests investigated here were as follows, *hPDT*: haplotype-based pedigree disequilibrium test; *hPDT-t*: haplotype-based pedigree disequilibrium test with 30% training data; *maxV*: maximal rare variant test; *vPDT*: variant-based pedigree disequilibrium test; *vPDT-t*: rare variant based pedigree disequilibrium test with 30% training data. We evaluated the effects of the proportion of causal variants (*d%*) and investigated the effects of the proportion of variants that increase risks (*r%*), and also investigated the effects of LD on power.

### Type I error rates

As shown in Table 1, the type I error rates were acceptable for all six tests at the nominal level of 0.05. The *hPDT-t* and *vPDT-t* tests were slightly conservative at type I error rates of 0.035 and 0.041, respectively. For these two tests, the weight of a haplotype/variant was set to be zero if it failed to get a significant value of test statistic on training data set as shown in Equation (10). Due to the rarity of variants/haplotypes and limited sample size, under the null hypothesis, many zero weights were observed in our simulation though *μ* was set to be 1.04 not as strict as 1.28 used by [17, 28]. Approximately 20 rare variants were tested using the simulated data (Table 1).

**Table 1:** **Type I errors and power on effect of the proportion of causal SNVs (** ***d%*** **) under significance level of 0.05**

			# of variants	***maxH***	***hPDT***	***hPDT-t***	***maxV***	***vPDT***	***vPDT-t***
Type I error	*d%*=0	*r%*=0	21.82(sd=4.63)	0.053	0.050	0.035	0.045	0.052	0.041
Power	*r%*=1	*d%*=0.5	23.02(sd=4.90)	0.456	0.906	0.275	0.465	0.894	0.326
		*d%*=0.4	22.63(sd=4.64)	0.373	0.734	0.230	0.396	0.744	0.242
		*d%*=0.3	22.65(sd=5.11)	0.316	0.515	0.163	0.306	0.525	0.177
		*d%*=0.2	22.31(sd=4.56)	0.261	0.265	0.096	0.251	0.272	0.132
		*d%*=0.1	21.89(sd=4.65)	0.148	0.090	0.073	0.154	0.083	0.065

Note: (1) “# of variants” indicates the mean and standard deviation (sd) values of the number of SNVs in a 20Kb test region on 1000 replicates (10 permutations for each replicate); (2) *d%* is the proportion of rare variants in the causal region to be causal; (3) *r%* is the proportion of causal variants increase risk.

*Abbreviations*: *maxH*: maximal haplotype test; *hPDT*: haplotype-based pedigree disequilibrium test; *hPDT-t*: haplotype-based pedigree disequilibrium test with training data; *maxV*: maximal rare variant test; *vPDT*: variant-based pedigree disequilibrium test; *vPDT-t*: rare variant based pedigree disequilibrium test with training data.

### Effect of the proportion of causal SNVs (d%)

We further evaluated the analytical power and the effects of the proportion of causal variants (*d%*). The proportion of variants that increased risks, *r%*, was fixed at 100%, which means that all variants within the causal variants increase disease risk. Table 1 shows that the haplotype-based tests obtained similar power to the corresponding rare variant based tests. The *hPDT* and *vPDT* provided more power than the other four tests, and the *hPDT-t* and *vPDT-t* tests were least powerful, and the performances of *maxH* and *maxV* tests fell in the middle.

### Effect of the proportion of variants that increase risks (r%)

The effects of the proportion of variants that increase risks (denoted by *r%*) were also investigated. The proportion of causal variants, *d%*, was fixed at 50%, which means that half of the rare variants in the causal region were causal. Similar to results presented in Table 1, the haplotype-based tests in Table 2 obtained similar power to the corresponding rare variant based tests. When *r%*=1 and 0.8, the *hPDT* and *vPDT* provided highest power than the other four tests, and the *hPDT-t* and *vPDT-t* were least powerful, and the *maxH* and *maxV* tests were in the middle. However, when *r%*=0.5 and 0.2, the *maxH* and *maxV* tests were most powerful when the joint effects were masked by noise from either causal variants with opposite direction (50% and 80%, respectively) or non-causal variants (50%). When *r%*=0.05, except the *hPDT-t* and *vPDT-t* tests obtained power of ~30%, all other four tests have no power to detect association under this condition.

**Table 2:** **Power and effect of the proportion of positive causal SNVs (** ***r%*** **) under significance level of 0.05**

Power		# of variants	***maxH***	***hPDT***	***hPDT-t***	***maxV***	***vPDT***	***vPDT-t***
*d%*=0.5	*r%*=1	23.10(sd=5.04)	0.439	0.882	0.255	0.463	0.879	0.313
	*r%*=0.8	22.21(sd=4.87)	0.390	0.617	0.229	0.390	0.598	0.238
	*r%*=0.5	21.60(sd=4.84)	0.294	0.161	0.174	0.293	0.152	0.199
	*r%*=0.2	20.93(sd=4.68)	0.167	0.089	0.087	0.158	0.095	0.090
	*r%*=0.05	20.17(sd=4.72)	0.045	0.288	0.048	0.044	0.293	0.062

Note: (1) “# of variants” indicates the mean and standard deviation (sd) values of the number of SNVs in a 20Kb test region on 1000 replicates (10 permutations for each replicate); (2) *d%* is the proportion of rare variants in the causal region to be causal; (3) *r%* is the proportion of causal variants increase risk.

*Abbreviations*: *maxH*: maximal haplotype test; *hPDT*: haplotype-based pedigree disequilibrium test; *hPDT-t*: haplotype-based pedigree disequilibrium test with training data; *maxV*: maximal rare variant test; *vPDT*: variant-based pedigree disequilibrium test; *vPDT-t*: rare variant based pedigree disequilibrium test with training data.

### Effects of LD under special haplotype setting

The effects of LD were investigated under the haplotype setting with LD and the results were shown in Table 3. Under the first *β* setting (*β*_1_ = *β*_4_ = 3, *β*_5_ = *β*_6_ = *β*_7_ = *β*_8_ = − 3), since the cumulative effect sizes *β*_*s*_ on each single rare variant were added to zero, the haplotype-based tests outperformed the rare variants based tests. Specially, *maxH* obtained the highest power (0.589) due to the gain from the first and fourth haplotype with significant positive effect sizes. While the *hPDT* and *hPDT-t* tests were less powerful due to the fact that the positive and negative effects carried by different variants may cancel each other out. The training data provided good estimates on the direction of weights due to strong effect of the first and fourth haplotypes, therefore, *hPDT-t* (power = 0.394) was more powerful than the *hPDT* (power = 0.114) test.

**Table 3:** **Power and effects of LD under special haplotype setting**

β settings	***maxH***	***hPDT***	***hPDT-t***	***maxV***	***vPDT***	***vPDT-t***
(1) *β*_1_ = *β*_4_ = 3, *β*_5_ = *β*_6_ = *β*_7_ = *β*_8_ = − 3	0.589	0.114	0.394	0.158	0.110	0.065
(2) *β*_1_ = *β*_4_ = 3, *β*_5_ = *β*_6_ = 1, *β*_7_ = *β*_8_ = − 3	0.541	0.088	0.331	0.505	0.258	0.168
(3) *β*_1_ = *β*_2_ = *β*_3_ = *β*_4_ = 2	0.488	0.412	0.230	0.691	0.616	0.391

Note: (1) Other βs are zero if not specified under each β setting; (2) For fifteen haplotypes, 1010000000, 0110000000, 1001000000, 0101000000, 1000000000, 0100000000, 0010000000, 0001000000, 0000100000, 0000010000, 0000001000, 0000000100, 0000000010, 0000000001, 0000000000, the first fourteen rare haplotypes have equal frequencies of 0.01 and the last common haplotype has frequency of 0.86; (3) Simulation results are based on 1000 replicates (10 permutations for each replicate).

*Abbreviations*: *maxH* maximal haplotype test, *hPDT* haplotype-based pedigree disequilibrium test; *hPDT-t* haplotype-based pedigree disequilibrium test with training data, *maxV* maximal rare variant test, *vPDT* variant-based pedigree disequilibrium test, *vPDT-t* rare variant based pedigree disequilibrium test with training data.

Under the second *β* setting *β*_1_ = *β*_4_ = 3, *β*_5_ = *β*_6_ = 1, *β*_7_ = *β*_8_ = − 3), the cumulative effect sizes *β*_*s*_ on the first two variants were greater than 3, *maxH* remained to be most powerful (0.541) though the *maxV* test gained similar power (0.505). As same as under the first *β* setting, the *hPDT-t* outperformed *hPDT* since the risk, protective and natural haplotypes were well predicted by the training data set (using Equation (10)) when haplotypes have both positive and negative effects and these effects were sufficiently strong (for example, the haplotypes take the value of 3 and −3) to be detected under the first and second β settings.

Under the third *β* setting (*β*_1_ = *β*_2_ = *β*_3_ = *β*_4_ = 2), the cumulative effect sizes (*β*_*s*_) on the first four variants were 4, which were greater than the haplotype effect sizes. Therefore, the rare variant based tests gained more power than the haplotype-based approaches (in the order of *maxH < maxV*, *hPDT < vPDT* and *hPDT-t < vPDT-t* on power). Under this parameter setting, as all effect sizes were in same direction on both haplotypes and rare variants, the methods based on training data had not gain any power than the methods without training data (*hPDT-t* < *hPDT* and *vPDT-t* < *vPDT* on power). The *maxH* test obtained was similar power under three *β* settings.

Based upon the simulation results as shown in Table 3, the power achieved using these two types of methods, haplotype-based pedigree tests vs. rare variants based pedigree tests, depending on which one had greater contribution on disease between haplotypes and rare variants. To detail, the haplotype-based pedigree tests had enhanced statistical power when the disease of interest was mainly contributed by rare haplotypes (with multiple rare alleles), and the rare variants based tests were more powerful when disease was independently caused by rare variants. For other situations when disease was caused both by haplotypes and rare variants with similar effect sizes, these two approaches offered similar power in testing for association.

When we used *Forsim* to simulate pedigrees that could be considered to be close to the real human population. In a real population, the haplotype frequencies were small on those haplotypes with two or more than two rare variants. For instance, the haplotype frequency was less than 0.0004 for a haplotype with two rare variants with equal to MAF of 0.02 when there is no LD between these two rare variants (i.e. Hardy-Weinberg Equilibrium). Under this circumstance, both haplotypes and rare variants had comparable contribution to disease since the causal variants were selected randomly, therefore, the two approaches had similar power in testing for association as shown in Tables 1 and 2.

Having demonstrated the power of the *hPDT* approach, we would like to note that the simulated genetic models were conducted under a few optimistic assumptions, and these assumptions may inflate the estimated power. In reality, hunting for rare and causal variants is known as a difficult task. Multiple variants from different genes can contribute to the genetic etiology of complex diseases, and looking at one gene at a time may cause missingness of causal variants. This is viewed as a limitation of our study. In future studies, we will broaden the scope of our research efforts by applying gene enrichment approaches to identify relevant molecular pathways involved in the occurrence of human diseases in multiple affected individuals in pedigrees. The extent of identity by descent (IBD) sharing will direct us where to look for the causal variants.

From a biological standpoint, several haplotype mutations are more likely to alter amino acid coding and therefore lead to a greater joint effect influencing traits of interest than a single amino acid change caused by a single mutation [29]. The availability of WGS or whole exome sequencing (WES) technology enables keen researchers to explore the role of rare individual variants. It is important to note that the idea of joint effects of a disease haplotype could again be used in next generation sequencing. One could argue that the haplotype-based approach may more effectively identify disease-relevant haplotypes. Furthermore, this haplotype-based approach may provide some information regarding un-genotyped causal variants because of its potential to identify the interaction between two or more rare variants.

Finally, we acknowledge that, ideally, the feasibility of a newly developed test should be assessed using a real sequencing dataset. However, due to the rarity of sizable collections of family genomes in related individuals, we were unable to compare the above six tests in a real dataset. As an alternative, we applied those methods to a gene-based data set which contains a good portion of rare variants (10%) generated by the Illumina iSelect IBC Chip.

### Application to Framingham heart study data

To prove the feasibility of the *hPDT*, we evaluated the performance of our test using the Framingham Heart Study (FHS) data set (downloaded from dbGAP study: phs000007.v20.p8) and we also used the *vPDT* to conduct the analysis on the same data set. The FHS cohort includes 6911 individuals (3726 females and 3185 males in 1211 pedigrees) in three generations; their mean and standard deviation (sd) age is 40.9 and 9.1, respectively. Forty-seven thousand and thirty-seven SNPs were genotyped in the Illumina iSelect IBC Chip. After quality control, there remained 4293 rare SNPs with MAFs≤0.05 in 780 genes in the FHS cohort, and we analyzed these SNPs using gene-based methods. The qualitative trait of hypertension was defined as systolic blood pressure (SBP) ≥ 140 or diastolic blood pressure (DBP) ≥ 90 or taking medicine for hypertension.

As noted in Table 4, for both *hPDT-t* and *vPDT-t* tests, the p-values were taken as 1 when all single haplotype/variant in training data set failed to pass the association test and all weights are set to be zero. Based upon the p-values shown in Table 4, the haplotype-based pedigree tests achieved better statistical power than the rare variants based tests. In reality, both positive and negative effects are expected to exist within the same gene and these variants with the effects of opposite directions might cancel each other out. When haplotypes show greater effect size, the haplotype test is easier to be detected. Therefore, it is worth to employ haplotype based tests to detect association caused by rare variants as well as variant based tests.

**Table 4:** **The significant genes from FHS study with p-values < 0.001 at any test**

gene	Chromosome	Nsnp	***maxH***	***hPDT***	***hPDT-t***	***maxV***	***vPDT***	***vPDT-t***
PLG	chr6	10	0.02498	**1.05E-05**	0.08819	0.43656	0.50666	1
TFPI	Chr2	18	0.00099	0.00146	9.76E-05	0.45654	0.88330	1
TNFRSF4	chr1	7	1	0.71721	0.00056	0.64336	0.37994	1
TGFB3	chr14	10	0.01998	0.00060	1	0.16284	0.62846	1
IL1R2	chr2	21	0.00200	0.19275	0.00067	0.16783	0.91804	1
MMP16	chr8	9	0.09790	0.00090	1	1	0.31731	1
LRP2	chr2	21	0.00100	0.61149	0.00091	0.03197	0.14087	1
LEP	chr7	18	0.05395	0.00093	0.06325	0.66533	0.35130	1

*Abbreviations*: *maxH* maximal haplotype test, *hPDT* haplotype-based pedigree disequilibrium test, *hPDT-t* haplotype-based pedigree disequilibrium test with training data, *maxV* maximal rare variant test, *vPDT* variant-based pedigree disequilibrium test, *vPDT-t* rare variant based pedigree disequilibrium test with training data.

As shown in Table 4, the top ranked eight genes were shown in FHS study with p-values < 0.001 on the haplotype-based or variant-based test. Out of all genes examined in the FHS study, PLG gave the most significant p-value (1.05E-05) and remained significant under Bonferroni correction significance level of 6.4E-05 (calculated by 0.05/780). Although we did not find evidence for direct association between SNPs in PLG and hypertension, we found a record for a predictive role of PLG for carotid artery disease risk [29]. LRP2 encodes the low density lipoprotein receptors-related protein 2, and has been implicated in regulation of signaling pathway, which is involved in diverse biological processes including lipid metabolism. Therefore, LRP2 can be viewed as a putative candidate for disorders related to lipid metabolism [30]. Obesity is closely associated with increased morbidity and mortality caused by cardiovascular diseases, diabetes, and hypertension [31], however, at the time being, our software cannot accommodate quantitative factors (such as height).

## Conclusion

Here, we introduced a few novel haplotype-based PDT approaches for detecting rare variants using a weighted combination of rare haplotypes in pedigree data. As we reviewed earlier, in genetic studies, the haplotype structure is expected to carry important information. It has been found that the haplotype information gained from family members may result in more reliable estimates of the phase of haplotype. Moreover, as high-throughput sequencing is becoming widely used, the haplotyping methods can borrow some partial phase information in sequence reads to increase the accuracy of haplotypes estimated from genotype data [32].

In addition, we sought to develop a method that could be used in family designs for following reasons: 1) family-based haplotype methods are more robust than case–control-based methods; and 2) if a rare haplotype is observed in a family, the haplotype may be enriched more frequently than in population-based designs. Therefore, the family-based haplotype design would be expected to be a more powerful of association between a collection of multiple rare variants and disease-relevant phenotypes.

Both the *hPDT/hPDT-t* and *vPDT/vPDT-t* approaches—drawn from the same PDT framework—were used in different sets if simulations. Further, *maxH* and *maxV* were also proposed to detect the signal of a single haplotype/variant. Such a comparison is the most intuitive way to assess whether the power increase are caused by the strategy used. Our simulations revealed that the haplotype-based approaches may achieve more power when the disease was more likely caused by causal haplotypes as shown in Table 3. On the other hand, as shown in the simulation using forward evolutionary process, the haplotype-based methods and the rare variants based methods performed similarly when the disease was caused by both causal haplotypes and causal variants as shown in Tables 1, 2 and 3. With the advent of increasingly reduced WGS costs, future comprehensive comparison studies will be conducted to compare all rare variant detection methods on multiple sequencing data sets with pedigrees structure.

To benefit potential users, all six tests (*maxH*, *hPDT, hPDT-t*, *maxV*, *vPDT* and *vPDT-t*) have been implemented in the “rvHPDT” software coded by R language.

### Web resources

MERLIN: http://www.sph.umich.edu/csg/abecasis/merlin/tour/haplotyping.html

The rvHPDT package has been submitted to the comprehensive R archive network (CRAN): http://cran.r-project.org/.
